# Patricia E. Bath: A Luminary in Ophthalmology

**DOI:** 10.7759/cureus.65781

**Published:** 2024-07-30

**Authors:** Vihaan Anumula, Pooja Kanyadan, Chitra Kanyadan

**Affiliations:** 1 Biology, Wheeler High School, Marietta, USA; 2 Clinical and Translational Sciences, University of Rochester, Rochester, USA; 3 Internal Medicine, Wellstar Health System, Marietta, USA

**Keywords:** global healthcare, innovation in ophthalmology, ophthalmic surgery, access to healthcare, blindness prevention, preventive ophthalmology, community ophthalmology, cataract

## Abstract

Renowned ophthalmologist and inventor Dr. Patricia Bath made ground-breaking contributions to medicine. After seeing that blindness was more common among Black patients, she focused much of her career on improving access to eye care for underserved communities. Bath’s most important contributions to her field of ophthalmology were the inventions of community ophthalmology and the laserphaco probe. Community ophthalmology improves access to eye care by combining treatment, education, and preventive care into a coherent program targeted toward serving underprivileged populations. The laserphaco probe combines laser fragmentation with ultrasonic removal, which greatly improves the accuracy and safety of cataract surgery. Laser technology’s precision also reduces the possibility of surgical mistakes, thus increasing success rates and improving the general results. Apart from improving the quality of life for many people, Dr. Bath’s inventions established a new benchmark in blindness prevention and cataract therapy, thus guiding modern ophthalmology and ocular surgery.

## Introduction and background

**Figure 1:**
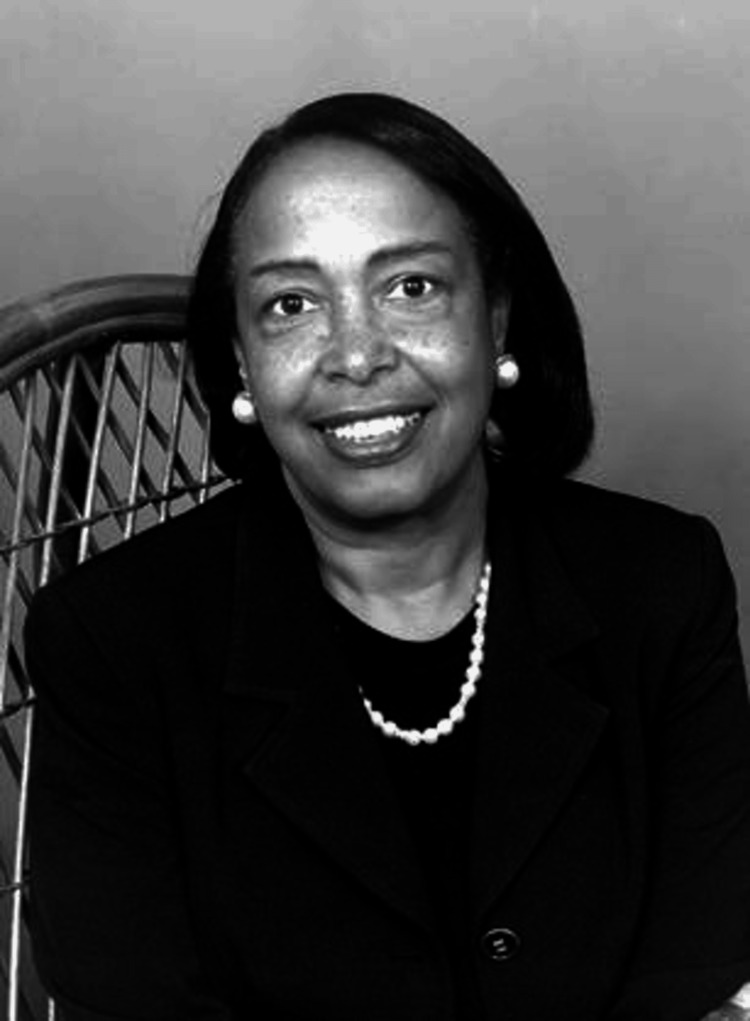
A seated portrait taken of Dr. Patricia Bath This image is available through the National Library of Medicine. As a work of the U.S. federal government, the image is in the public domain [1].

Dr. Patricia Bath, a renowned ophthalmologist and laser scientist, made ground-breaking contributions to medicine by improving the quality of and access to eye care for all (Figure 1) [1]. Focusing on ophthalmology, Bath’s career was driven by her relentless quest for improvement in eye care, especially for the underserved [1,2]. Her intense interest in the field of prevention of blindness and visual acuity drove her work outside of clinical settings into study, advocacy, and invention [3,4]. In 1976, Bath pioneered the concept of community ophthalmology, which uses public health, community medicine, and clinical practice to prevent and treat blindness in underprivileged areas [3]. She started her first program in Harlem Hospital, New York, NY, and her outreach helped prevent blindness in thousands of people who would have otherwise not been diagnosed and treated [3]. Continuing her dedication to preventing avoidable blindness, Dr. Bath patented the laserphaco probe in 1986, which uses precise laser technology to gently and precisely remove cataracts from patients [5,6]. Her contributions not only have advanced medical technology but also opened the medical and scientific fields to the next generations of women and underprivileged populations globally [1,4].

## Review

Early life and career 

On November 4, 1942, in Harlem, New York, housewife Gladys Bath and Trinidadian motorman Ruper Bath welcomed Patricia Bath into the world [1,4]. When Bath was young, her mother presented her with many books and a chemistry set, which sparked her scientific curiosity [2,3]. Bath had many hardships as an African American girl with aspirations to become a physician, including sexism, racism, and poverty. Growing up, she did not know any female physicians, and though she aspired to become a surgeon, she knew it was a male-dominated profession [1]. However, Dr. Albert Schweitzer’s humanitarian work in the Congo with people infected with leprosy and her relationship with her family physician, Dr. Cecil Marquez, fueled her ambition to pursue medicine [1,3]. 

Dr. Bath attended Charles Evans Hughes High School in Manhattan. She was an exceptional student who received a National Science Foundation grant to attend the Summer Institute in Biomedical Science at Yeshiva University, New York, in 1959, where she studied the relationship between cancer, nutrition, and stress [2]. During her studies, Bath formulated an equation to predict cancer cell growth. Impressed by her work, her mentor, Dr. Robert O. Bernard, added her findings to a paper that he presented in 1960 at an international medical conference in Washington, D.C. Bath studied chemistry and physics at Hunter College in New York and graduated in 1964. Although African American students were excluded from many medical schools, she was admitted to Howard University Medical School, Washington, D.C.; however, her family did not have the money to send her [1]. She recounts that her mother scrubbed floors so that she could attend medical school, from which she graduated with honors in 1968 [1,2,4]. She was the first African American resident at New York University and finished her medical training in 1973. She completed a fellowship in corneal and keratoprosthesis surgery in 1974 [1,5]. Her preparation for a career in medicine came from her training, which emphasized research and clinical practice.

As a young intern at Harlem Hospital and the eye clinic at Columbia University, New York, Dr. Bath observed major differences in eye care between Black and White patients [3,6]. After seeing that blindness was more common among Black patients due to a lack of access to eye care, she created “community ophthalmology,” an interdisciplinary health management approach that used public health, community medicine, and clinical practice to provide eye care to underserved communities [3,6,7]. Community ophthalmology has been recognized as a powerful tool for reducing disparities in eye health, and its use is being expanded all over the world [8]. Apart from her clinical practice, Bath’s vision to improve access to eye care led her to lobby for better care and educate others, and she did so by co-founding the American Institute for the Prevention of Blindness in 1976 [4,6]. This global nonprofit organization was centered around providing all people with primary eye care.

Bath faced several challenges as an African American woman. As the first African American resident in ophthalmology at New York University (1970-1973), she encountered prejudice and discrimination regularly [1,4]. She overcame these obstacles and went on to have a successful career, improve working conditions, and become the first female faculty member in the Department of Ophthalmology at the University of California, Los Angeles (UCLA), CA [1,2]. In 1983, she was the first woman in the United States to be appointed chair of an ophthalmology residency training program at Drew-UCLA [1]. In 1981, Bath developed the idea for the laserphaco probe, a cataract removal tool employing laser technology. This development sought to minimize the time, risk, and discomfort related to cataract surgery [4,5]. Her research and development of the laserphaco probe were limited in the United States due to racism and sexism; however, Bath refused to be limited by these “glass ceilings” and took her research to Europe [1]. She persisted in the face of doubt and technical challenges, traveling all throughout Europe to get the state-of-the-art laser tools required for her studies. Her research was accepted at the Laser Medical Center of Berlin, West Germany; the Rothschild Eye Institute of Paris, France; and the Loughborough Institute of Technology, Loughborough, UK [1]. Her patent for the laserphaco probe, which transformed cataract surgery and greatly improved patient care, was awarded in 1988 [1, 5].

Community ophthalmology

After observing clear racial and economic disparities between the patient communities at Harlem Hospital and the eye clinic at Columbia University, Dr. Bath conducted a retrospective epidemiological study that found that Black patients had double the rate of glaucoma and blindness compared to White patients due to lack of access to eye care [6,7,9]. As a result of her findings, Dr. Bath pioneered the discipline of community ophthalmology in 1976 [6]. 

Community ophthalmology is an interdisciplinary health management strategy using public health, community medicine, and clinical practice to prevent and treat blindness in underprivileged areas [7,10]. These programs improve access to eye care by combining treatment, education, and preventive care into a coherent program targeted toward serving underprivileged populations [11,12]. Specifically, community ophthalmology focuses on the prevention of eye disease before it occurs via screening, prevention of blindness if the disease has occurred via safe surgical procedures and treatment, and education at various levels [13]. These initiatives require regular training of primary care workers, adequate referral systems, and education aimed at making people aware of eye diseases, their risk factors, and eye healthcare programs available in their community [11, 13]. 

Dr. Bath started her first community ophthalmology project at Harlem Hospital in New York [4]. Dr. Bath trained volunteers as community eye care workers to provide primary eye care to chronically underserved populations [9]. They visited senior centers and daycare programs to test patient vision and screen for cataracts, glaucoma, and other serious eye conditions that could result in blindness [9,10]. Through this community outreach, Dr. Bath was able to give underserved populations a better chance at preventing higher rates of blindness by screening for disease early on and treating existing diseases before blindness developed [10]. Her outreach prevented blindness in thousands of people who would have otherwise not had any access to diagnostic and treatment services [3].

Dr. Bath’s principles behind community ophthalmology ensure that the general public is aware of warning signs of eye disease and that vision screening is available to all, as methods to check for visual acuity and intraocular pressures (indicators of developing blindness and glaucoma, respectively) are simple procedures that do not require elaborate training or equipment, which will improve eye health outcomes for systemically underserved populations [7]. While support from governments and large public health organizations is necessary for blindness prevention to be successful, there must be collaboration with non-government community organizations and locally trained health personnel, who can connect with their communities to deliver eye care, change their health behaviors, and achieve a “health revolution” [13]. With this in mind, the World Health Organization (WHO) and many countries around the world have embraced Dr. Bath’s principles in their efforts to prevent avoidable blindness. The WHO established the Programme for the Prevention of Blindness in 1978, and the first principles were published in 1979 [14]. This publication echoed Dr. Bath’s methods used in Harlem; the first line of prevention is to identify communities with high prevalence of avoidable blindness, followed by treating known causes of blindness, such as malnutrition or cataracts, and strengthening existing ophthalmic services, such as by improving referral systems and training for primary care workers [14,15]. As stated earlier, community ophthalmology makes these services possible [7,11-13]. By 1995, The WHO Programme for the Prevention of Blindness was adopted in 27 countries in Africa, some countries in the Americas and Eastern Mediterranean region, five countries in Southeast Asia, with a particular focus on India, and eight countries in the Western Pacific region [15].

One of the most notable programs for the prevention of blindness is in India, where, as of 2022, there are an estimated 4.95 million people who are blind [11,15,16]. By means of extensive screening and appropriate surgical interventions, community ophthalmology initiatives have been quite helpful in early detection and in lowering the rate of avoidable blindness in India [8,14]. These initiatives include general eye screening camps, fixed facilities (primary eye care centers and vision centers), mobile eye care units, school eye screening, diabetic retinopathy screening, teleophthalmology, and cataract surgical camps [11]. In remote locations where access to conventional healthcare facilities is restricted, these initiatives have especially been successful [15]. 

In sub-Saharan Africa, community-based eye care initiatives have similarly improved access to treatment for disorders such as trachoma, therefore boosting the quality of life for many people [15]. Mobile clinics and local health workers have been especially important in these areas in reaching far-off communities and offering much-needed treatment [14,15]. 

Community ophthalmology was especially useful during the COVID-19 pandemic, as it allowed for high-quality eye care to remain available without placing patients at risk of contracting the virus. A study in London, UK, assessed the number of patients treated by the Rapid Access Clinic (RAC) and the community Acute Primary Care Ophthalmology Service (APCOS) during COVID-19 in a region serving 1.2 million people [17]. Their findings showed that community ophthalmology services delivered by registered therapeutic optometrists can safely and efficiently treat and manage the majority of ophthalmology cases, which reduced the need for visits to emergency eye clinics in hospital eye departments during the pandemic [17]. The extra capacity provided by community ophthalmologic settings could be utilized in the future to direct general, non-emergent, or low emergency cases to the community settings, which would decrease hospital burden and wait times, especially during crises like the COVID-19 pandemic.

The laserphaco probe

**Figure 2:**
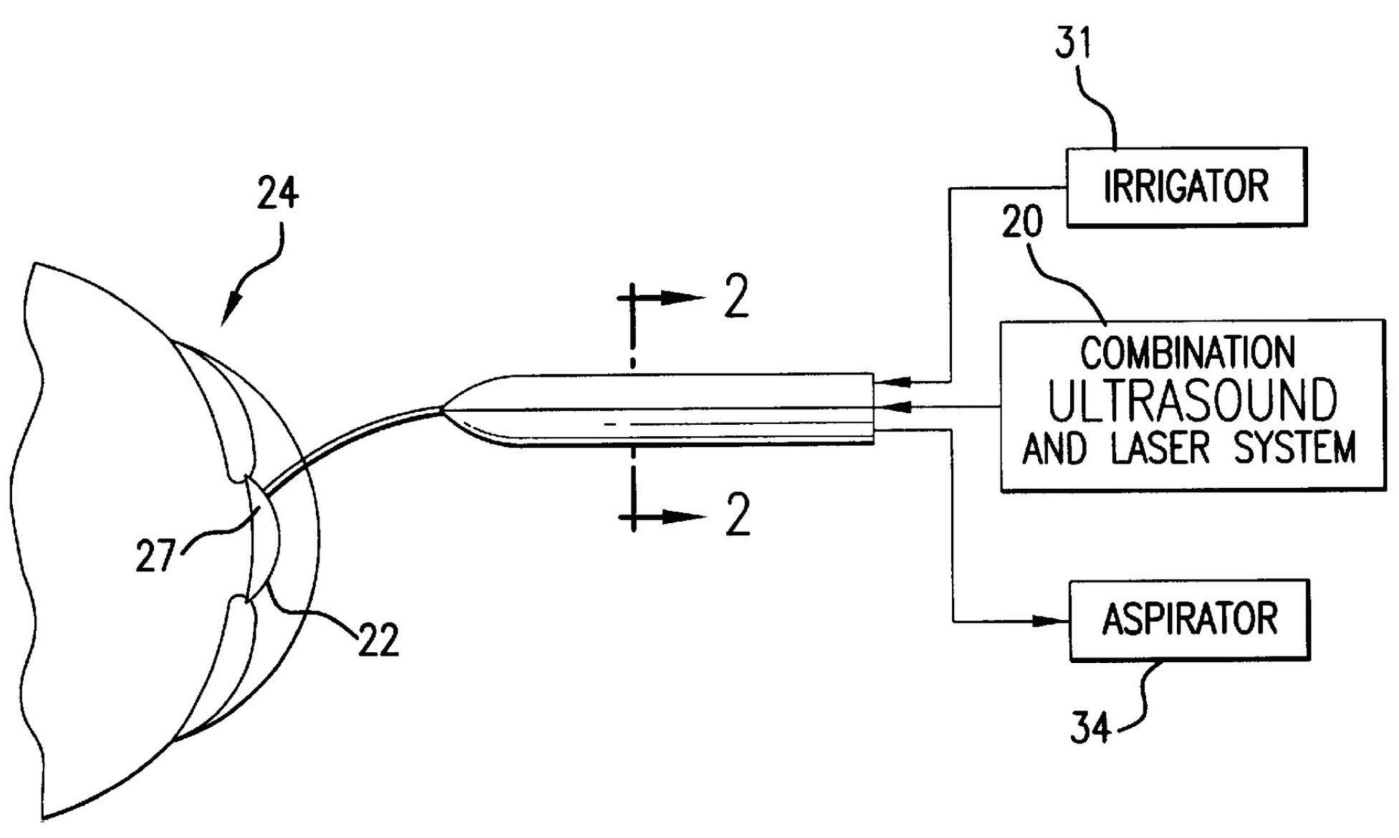
A diagram of the laserphaco probe design Numbers 20, 22, 24, and 27 correspond to the following, respectively: the ultrasound and laser portions of the probe, which are used to break apart the cataract; the cataract lens; the cornea of the eye; and the distal endface of the probe in contact with the cataract lens. This image is available through the U.S. Patent and Trademark Office. As a work of the U.S. government, this patent has been published for public viewing, and as such, the patent figures are available in the public domain [18].

Continuing her work in the prevention of avoidable blindness, Bath invented the laserphaco probe (Figure 2) [1-6]. Used to cure cataracts, a common eye ailment that causes lens clouding and is one of the leading causes of blindness, this creative instrument revolutionized cataract surgery by offering a more accurate, safer, and more efficient approach for cataract removal [6]. Dr. Bath understood the need for a more efficient treatment that could become widely available and lower the dangers related to conventional cataract operation [4]. Her aim was to design a tool that would increase access to sight-restoring operations in addition to enhancing surgical results [3].

The laserphaco probe operates by a methodical procedure combining exact mechanical motions with laser technology. The center of the probe is a fiber-optic laser, which sends laser and ultrasonic energy to the cataract lens. This laser is nested inside an irrigation sleeve, which contains irrigation and suction tubes and provides constant cooling and waste removal during the operation [18]. The surgery starts with the surgeon making a small incision in the eye to reach the lens affected by a cataract [5]. First, the laser generates extremely concentrated light pulses that break the cataract into pieces. This fragmentation is essential since it makes the damaged lens easier to remove without compromising the surrounding ocular structures [4,5]. Additionally, well-chosen parameters to maximize lens fragmentation help reduce thermal injury to nearby tissues while generating the laser energy required for photodisruption [18].

The ultrasonic tip of the probe comes into use once the cataract fragments. High-frequency sound waves produced by the ultrasonic transducer break the cataract lens into even smaller pieces that are less than 1 mm large [18]. Using the integrated vacuum system of the probe, the broken bits are then softly aspirated (suctioned) from the eye [1,4]. The probe keeps a regulated environment inside the eye throughout this operation, which limits the dispersion of lens particles and guarantees a clear surgical field [1,18]. Additionally, because the particles produced by fragmentation of the lens are so small (less than 1 mm), the probe itself is very small, which allows for the surgical incision site to be small and allows for faster healing of the wound [18]. After the cataract is removed, a replacement lens can be inserted, restoring the patient’s sight [5]. Combining laser fragmentation with ultrasonic removal in the laserphaco probe greatly improves the accuracy and safety of cataract surgery [2].

Ophthalmology has considerably changed since the arrival of the laserphaco probe. Before its development, cataract surgery used more intrusive manual extraction methods with longer recovery times, higher risks of complications, and more involved effort [5]. Through the laserphaco probe, the treatment became faster, safer, more effective, and minimally invasive. This has permanently changed cataract surgery; it has reduced postoperative discomfort, shortened surgical times, and reduced recovery time, which has made cataract treatment more available to the world [1]. Laser technology’s precision also reduces the possibility of surgical mistakes, thus increasing success rates and improving the general results. Although it is not used in routine practice, Dr. Bath’s invention improved the quality of life for many people and established a new benchmark in cataract therapy and modern ocular surgery.

Late career

Dr. Patricia Bath kept changing the medical industry and promoting healthcare for all in her later years. Even after she left the UCLA Medical Center in 1993, her dedication to increasing eye care availability never wavered [1]. Dr. Bath kept working toward multiple approaches to cure blindness. In 2000 and 2003, she obtained a patent for a combination of ultrasound and laser to treat cataracts [3]. Promoting the use of electronic communications to provide medical care to underprivileged and remote places, Bath also became a forerunner in telemedicine [19], and she was appointed to President Barack Obama’s panel for digital accessibility for the blind in 2009. Emphasizing the need for world health projects, she maintained telemedicine jobs at Howard University and St. George’s University in Grenada [19].

Bath’s induction into several halls of fame, including the International Women in Medicine Hall of Fame in 2001, solidified her legacy even more [1]. She remained a champion of science education and eliminated obstacles for women and minorities in the medical industry while still garnering honors for her work [2,4]. Bath’s contributions to world health have been long-lasting; her pioneering work in telemedicine and support of fair healthcare has opened the path for the next developments in medical accessibility [2].

## Conclusions

Dr. Patricia Bath enhanced the profession of ophthalmology with her development of community ophthalmology and the laserphaco probe, which transformed prevention strategies for blindness and made cataract surgery safer and more accessible. Global health has been permanently changed by her commitment to bettering eye care, especially for underprivileged areas, and her innovative work in telemedicine. Bath’s contributions to medicine go beyond her inventions, as she continuously promoted fair healthcare and eliminated many obstacles for women and minorities working in medicine, which opened the path for many future developments. Future generations of doctors will always find inspiration in her legacy.
